# Dietary Knowledge and Myths Vary by Age and Years of Schooling in Pregnant Mexico City Residents

**DOI:** 10.3390/nu12020362

**Published:** 2020-01-30

**Authors:** Reyna Sámano, Citlali Lara-Cervantes, Hugo Martínez-Rojano, Gabriela Chico-Barba, Bernarda Sánchez-Jiménez, Orly Lokier, María Hernández-Trejo, Juan Manuel Grosso, Solange Heller

**Affiliations:** 1 Departamento de Nutrición y Bioprogramación, Instituto Nacional de Perinatología, Secretaría de Salud, Ciudad de Mexico 11000, Mexico; ssmr0119@yahoo.com.mx (R.S.); gabyc3@gmail.com (G.C.-B.); pimpodhumo@gmail.com (J.M.G.); solhrouas@gmail.com (S.H.); 2 Coordinación de Nutrición. Universidad Cuauhtémoc, Aguascalientes 20116, Mexico; citlali.larac@hotmail.com; 3 Sección de Posgrado e Investigación, Escuela Superior de Medicina del Instituto Politécnico Nacional, Plan de San Luis y Díaz Mirón s/n, Colonia Casco de Santo Tomas, Delegación Miguel Hidalgo, Ciudad de Mexico 11350, Mexico; 4 Coordinación de Medicina Laboral, Instituto de Diagnóstico y Referencia Epidemiológicos (InDRE) “Dr. Manuel Martínez Báez”, Secretaría de Salud, Ciudad de Mexico 01480, Mexico; 5 Escuela de Enfermería, Universidad Panamericana, Ciudad de Mexico 03920, Mexico; 6 Subdirección de Investigación en Intervenciones Comunitarias, Instituto Nacional de Perinatología, Secretaría de Salud, Ciudad de Mexico 11000, Mexico; emiberna20@yahoo.com.mx; 7 Departamento de Nutrición, Universidad del Valle de México-Chapultepec, Ciudad de Mexico 11810, Mexico; orlylokier@gmail.com; 8 Departamento de Neurobiología del Desarrollo, Instituto Nacional de Perinatología, Secretaría de Salud, Ciudad de Mexico 11000, Mexico; maria.h.trejo72@gmail.com

**Keywords:** pregnancy in adolescence, breastfeeding, pregnant women, feeding behavior, Mexico, diet

## Abstract

Pregnancy is a stage in a woman’s life when she is more open to receiving health advice, especially related to diet. However, women are often caught between receiving scientifically unfounded myths and concrete empirical knowledge. Culturally perpetuated myths may be acted upon more than knowledge, but research on these concepts, especially in the Americas, is scarce. This cross-sectional study aimed to describe the frequency of diet and nutrition myths and knowledge and describe the associated factors in pregnant mothers receiving care in Mexico City. A total of 695 pregnant adults and 322 pregnant adolescents participated in this study, in which they responded to a questionnaire on nutrition and diet myths, knowledge, and practice during pregnancy and breastfeeding. The myths were examined individually, but for the purposes of statistical analysis, a score was obtained. We compared means of variables that could be associated to myth and knowledge scores, then calculated linear and logistical regressions. Forty-six percent of participants had below the mean myth scores. Ninety-two percent of participants had a knowledge score below the mean. Age (β = 0.025, SE 0.007, 95% CI 0.011–0.040, *p* = 0.001) and years of education (β = 0.166, SE 0.024, 95% CI 0.119–0.213, *p* = 0.001) explained the myth’s score, while age explained the knowledge score (β = 0.011, SE 0.020, 95% CI −0.032–−0.008, *p* = 0.002). We found that although most women reported not believing in the myths, they acted on them. The probability of practicing such myths as “You must eat for two during pregnancy” was associated with being an adolescent (OR 1.76, *p* = 0.001) and being married (OR 1.47, *p* = 0.007), “Not satisfying cravings leave a mark on the infant’s body” with being adolescent (OR 1.59, *p* = 0.003) and low socioeconomic level (OR 1.41, *p* = 0.038), “A frightened or angry mother should not nurse her baby” with being adult (OR 2.61, *p* = 0.004), and “Drinking *atole* or beer enhances breast milk production” with being single (OR 2.07, *p* = 0.001). The probability of not acting on some knowledge was associated with being an adolescent (*p* ≤ 0.003) and having a high school education or below (*p* ≤ 0.046). Almost all of our participants held at least one myth about nutrition and diet during pregnancy and breastfeeding; younger participants showed a higher frequency of holding myths. Years of schooling and age were associated with acting on myths and not acting on correct knowledge.

## 1. Introduction

Pregnancy is a stage in a woman’s life when she is more open to receiving health advice [1]. For instance, women are more prone to changing their nutritional habits based on what the clinicians, friends, or relatives recommend for a healthy pregnancy and their infant’s well-being. Indeed, women tend to adopt new nutritional habits during pregnancy and breastfeeding [2]. All cultures have unique nutritional habits, and numerous populations hold myths about differential dietary patterns during pregnancy [3]. A myth is any belief held to be true despite scientific information to the contrary and passed through the general population [4,5,6,7,8]. Myths can be detrimental to health, while on the other hand, we define knowledge as objective, scientific information related to beneficial effects for health [9]. During pregnancy, women receive nutritional information of both types—unfounded and possibly detrimental myths and scientific knowledge. However, myths are sometimes acted upon more than knowledge [1]. Research on dietary myths and knowledge in the Western hemisphere is scarce, as most research has been done in Africa and the Middle East [10,11].

Dietary habits have an impact on health in different stages of life; during pregnancy, these habits are associated with gestational weight gain and postpartum weight retention. While breastfeeding provides benefits for both a mother and her child—and most recognize the importance of breastfeeding [10,11,12,13]—there is a lack of knowledge on the specific nutritional value and immune effect of breast milk on an infant [13]. Moreover, misconceptions often exist about whether the quantity of milk produced by a woman is adequate to satisfy the needs of the newborn, especially in the first few days postpartum [11,12].

Most food practices during pregnancy and breastfeeding have been associated with the presence of myths and knowledge [14]. The proportion of these practices in different cultures may vary according to the age and educational level of pregnant women and mothers [15,16].

Myths and knowledge in the first 1000 days postpartum have an impact on health that still requires further investigation [17,18]. There is a clear need to provide dietary guidance during prenatal care and breastfeeding, and this guidance could be more effective if it takes myths into account. Because of the misconceptions and lack of information that exist surrounding diet in pregnancy and breastfeeding, our study aimed to describe the frequency of nutritional myths and knowledge in pregnant Mexico City mothers and compare these by age, years of schooling, income, marital status, and other socioeconomic variables. We hypothesized that pregnant women’s frequency of myths and knowledge about nutrition during pregnancy and lactation is associated with their age.

## 2. Material and Methods

### 2.1. Study Design and Subjects

A cross-sectional study was conducted with the participation of the *Instituto Nacional de Perinatología* (INPer) and the *Superior de Medicina* (Medical School) of the *Instituto Politécnico Nacional*, both located in Mexico City. INPer is a tertiary care center that offers prenatal check-ups to pregnant residents of the metropolitan area of Mexico City and neighboring states, most of whom are from low to lower-middle socioeconomic statuses.

Pregnant women who received prenatal medical care at the INPer between January 2014 and December 2017 were invited to participate. Sampling was non-probabilistic, and all consecutive patients who fulfilled inclusion and exclusion criteria were invited. Inclusion criteria included carrying a normal, healthy singleton pregnancy; absence of any chronic, degenerative, infectious or metabolic disease; delivering her child in the obstetrics care unit of the same hospital; and signing written informed consent. The exclusion criteria included consumption of alcohol, tobacco, or drugs during pregnancy; or being pregnant as a result of rape. All participants with incomplete information of variables were not used in the analysis. Participants between the ages of 11 and 19 were considered adolescents, and participants between the ages of 20–47 years were considered adults. The final sample included 1017 pregnant women, 695 adults and 322 adolescents.

We use a registration card to obtain all the necessary information from the medical record: gynecological data such as the age of menarche and the number of gestations, gestational age, and resolution of pregnancy. We also recorded socioeconomic levels (1 to 6), with 1 as the lowest and 6 as the highest. Finally, we recorded years of schooling, occupation, and marital status.

We obtained years of schooling, registered in years and categorized as “high school or below” and “university studies and higher.” Furthermore, we generated a variable to describe educational lag that measured if the adolescent’s real age was more than two years greater than the typical age in her current school grade. We defined educational lag in adult women as 15 or fewer years of schooling.

### 2.2. Data Gathering Methods

Myths and Knowledge

For the current study, a myth was considered a paradigm based on faith, without any scientific, objective, or empirical justification founded on public information. A myth is often assumed to be accurate and generates conformity. The fact that the myth is passed down from one generation to another gives it validity in a given family. Consequently, certain foods and eating habits are characterized in a way that cannot be questioned within the social circle professing the myths [7,8].

On the contrary, knowledge is generally accepted as the body of truths and tested actions accumulated over time in any civilization or country. In the present study, we adopt the concept of knowledge as the sum of objective, scientific-based information on nutrition, diet, and eating habits for pregnant and breastfeeding women [9].

We designed a structured questionnaire with closed questions; the questionnaire was prepared and available only in Spanish, as no non-Spanish-speaking women participated. First, the questionnaire was constructed, identifying two dimensions during pregnancy and breastfeeding: myths and knowledge. Many of these had already established by the scientific literature in the Mexican and Latin American populations [9,19,20,21,22,23,24].

The questions concerning myths were derived from comments continually heard from pregnant women during perinatal care and previously reported in two studies [19,20]. We included such myths as “you must eat for two during pregnancy,” “you can never get back to your ‘pre-baby weight’”, and “you must drink *atole* or/and beer to produce enough breast milk.” *Atole* is a traditional hot beverage in Mexico made of milk or water, cornstarch, and high amounts of sugar. Regarding beer, in Mexico, there is the myth that drinking beer during breastfeeding will increase breastmilk production, despite it being an alcoholic beverage [21,22,23,24]. The myths are were examined individually, but for the purposes of statistical analysis, a score was obtained. The myths are reference points used by pregnant and breastfeeding women to make dietary choices [25,26,27,28]. Each question about myths was scored with 1 point if the answer was “no” or “false” (correct) and zero if the answer was “yes” or “true” (incorrect).

The questions about nutrition and diet knowledge were, for instance, “caffeine consumption provokes premature birth,” “folic acid intake should begin before—not only during—pregnancy,” and “a mother requires an adequate increased energy intake during pregnancy and breastfeeding.” Each question about knowledge was scored with 1 point if the answer was “yes” or “true” (correct).

The practice of each myth and knowledge was measured using the following statements: “I have done that”, “I already do that”, “I would do that”, “I have not done that”, “I do not do that”, or “I would not do that”. There were seven questions about myths—four associated with pregnancy and three with breastfeeding—and ten questions about knowledge—six about pregnancy and four about breastfeeding. The ten knowledge statements were “Caffeine consumption provokes premature birth”, “Gestational diabetes increases future risk of type 2 diabetes”, “Folic acid intake should begin before—not only during—pregnancy”, “A healthy diet and lifestyle during pregnancy prevents future diseases in a child”, “The fetus receives vitamins, proteins, and minerals from what the mother eats”, “Obesity during pregnancy can cause hypertension and the risk of pre-eclampsia”, “A mother should consume about 3 L of water per day while nursing”, “The mother requires an adequate increased energy intake during pregnancy and nursing”, “During the first six months of life, breast milk is the only food a baby requires”, and “For successful breastfeeding, a baby must latch on to the nipple and use suction to feed”. The questionnaire was administered by professional personnel with experience in participating on research studies: one physician, two nutritionists, and one nurse. They were trained on how to apply the questionnaire.

The questionnaire was validated through a combination of evaluation by internal experts, laywomen, and external experts. First, we consulted a group of three experts in nutrition with more than five years of experience from INPer to verify the construct validity of the questionnaire items. Afterward, the questionnaire was administered to a pilot group of 30 women with similar characteristics to our study group. Their feedback was used to decide which questions were the most appropriate to be used in the final version of the questionnaire, and to ensure that the sentences and the order were understandable. Finally, the group of three researchers outside the INPer unaffiliated with the field of perinatology were consulted to corroborate the comprehensibility of the questions. After answering the myth and knowledge questions, a score was generated to measure participant knowledge. We applied and count only those elements that provided reliability and stability; leaving only seven items of the myths dimension and ten of the knowledge dimensions. Subsequently these same 30 participants were not part of the total sample studied. We validated the reliability through internal consistency, obtaining a Cronbach alpha coefficient of 0.82. We, therefore, concluded that the internal consistency, validity, and feasibility were at appropriate levels to be utilized.

### 2.3. Anthropometric Evaluation

The same trained personnel mentioned above performed the anthropometric measurements. Weight and height were obtained using the Lohman technique with participants wearing lightweight clothes. The Lohman technique consists of the standardization of the procedures for the different anthropometric measurements [29]. Participants’ Body Mass Index (BMI) was calculated by dividing their weight (in kilograms) by their height squared (in meters). Pregestational weight was self-reported and used to calculate pregestational BMI. For adults, the BMI was classified according to the guidelines of the Institute of Medicine (IOM, USA) into the categories of underweight, normal, overweight, and obese. For adolescents, the BMI was categorized according to the percentiles derived from the Centers for Disease Control growth charts for BMI for age and sex. Gestational weight gain was calculated by subtracting the pregestational weight from the final gestational weight at 38–40 weeks (maximum weight in kg). The weight of the newborn was examined on an infant scale (SECA 374, Baby and Mommy, 0.010 kg accuracy). The newborn length was measured with an infantometer (SECA 416, 0.1 cm accuracy). Finally, the medical record of the mother and baby was reviewed to obtain perinatal and sociodemographic data.

### 2.4. Ethical Aspects

Each participant was informed of the study’s objectives and the procedures involved, with emphasis on the voluntary nature of potential participation. All pregnant adults signed written informed consent, while pregnant adolescents signed written assent and their respective parent or guardian signed written informed consent.

The Ethics in Research Committee of the institution approved the research protocol with approval number 212250-49541. For confidentiality in data collection and analysis, each patient was identified by an identification number. After completing their participation in the study, each woman was given a brochure where all beliefs were corrected with a scientific-based explanation, and adequate information was provided. The brochure was generated using the same plain language as the questionnaire and targeted for optimal understanding in a group with lower health literacy.

### 2.5. Statistical Analysis

Frequencies and percentages were calculated for each myth and knowledge item. To facilitate bivariate analysis and binary regressions, the myth score was classified into two categories based on the median score obtained: 4 or fewer points was categorized as below the median level of belief in myths, while five or higher was categorized as above the median level of beliefs. For knowledge scores, the minimum score was 3 while the maximum score was 10; we, therefore, classified 3–6 points as below the median levels of knowledge, while 7–10 points were classified as above the median.

First, we used a Student’s t-test to compare the means of all continuous variables by above-average and below-average myth and knowledge scores. We also calculated Pearson’s chi-squared test to compare nominal variables. Then we compared means of maternal and neonatal variables according to the correct or not correct answer of each myth and knowledge using Student’s t-test. Linear regression models were made to obtain explanative variables of the myth and knowledge median scores. Interactions between factors and co-variables were sought using a univariate general lineal model. Finally, we performed binary logistic regression models to identify the variables associated with practicing the myths and not acting on knowledge during pregnancy and lactation. The regression models were adjusted for sociodemographic and gynecological variables. The data were analyzed in the 21st version of the SPSS statistical program for Windows (Armonk, NY, USA). Statistical significance was considered at *p* < 0.05.

## 3. Results

Of the 1017 participants in the study, 695 were adults and 322 were adolescents. Only 9% of this population did not believe any of the myths, and 46% of the participants had an above average myth score. Fewer than 1% (0.5%) thought all the myths correct (Table 1). On the other hand, in terms of knowledge, the vast majority of the participants had correct answers, and only 8% had below average score levels.

The mean knowledge score was 8 ± 1, with a range of 3–10, and an interquartile range of 8–9. Concerning myths score, we had mean of 4 ± 1, with a range of 0–7, and an interquartile range of 4–6.

**Figure 1:**
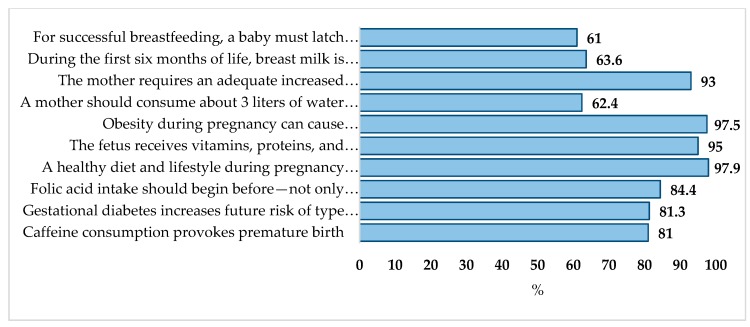
Distribution of participants that had correct knowledge, (%).

Figure 1 shows that, while the majority of the population reported correct knowledge for most categories, the statements with the lowest scores were, “Mothers requires an adequate increased energy intake during pregnancy and nursing”, “A mother should consume about 3 liters of water per day while nursing”, and “For successful breastfeeding, a baby must latch on to the nipple and use suction to feed”, with 63%, 62%, and 61%, respectively.

**Figure 2:**
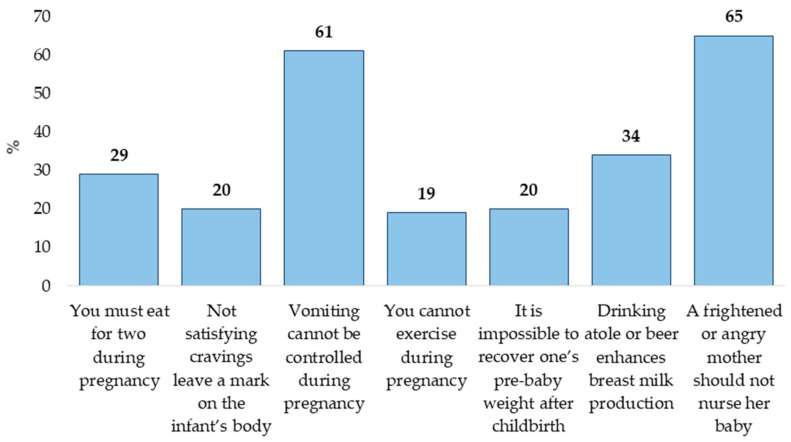
Distribution of participants that held myths.

Figure 2 demonstrates that “A frightened or angry mother should not nurse her baby” and “Vomiting cannot be controlled during pregnancy” were the most frequent myths.

Table 2 shows the statistical differences between almost all variables according to the above-average and below-average myths score. In contrast, there was no statistically significant difference in the questionnaire of knowledge with the same variables.

Table 3 shows maternal and neonatal variables associated with belief in seven myths of pregnancy and breastfeeding, we only present the variables that showed a *p*-value < 0.05. We found that the socioeconomic level was a major factor related to believing in the majority of the dietary myths of pregnancy and lactation. Two variables are matched in importance, intimately related to each other, age, and schooling. When the age is older, when they have more years of study, and a higher socioeconomic level, the chance of believing in most dietary myths of pregnancy and lactation is lower. The experience in maternity also seemed to have this association, because the greater the number of pregnancies, the lower the possibility of believing in certain myths. The last three columns would reflect the association of the belief of some myths about pregnancy outcomes. The birth weight was significantly higher in the children of those who do not believe in some myths. Perinatal, sociodemographic, and neonatal variables did not appear to explain any differences in knowledge.

Regarding pregestational BMI, adolescents had more low and normal weight compared with adults (12% vs. 4%, and 75 vs. 45%, respectively). On the other hand, adults had more overweight and obesity than adolescents (10 vs. 35%, and 3 vs. 16%, correspondingly). All comparisons were statistically significant (*p* = 0.001, Pearson’s chi-squared test).

Table 4 explains how age and years of education were associated with the myth score, with a low coefficient of determination (14.7%) but narrow confidence intervals. While only age was associated with knowledge score with a short accuracy interval and a very small coefficient of determination (0.5%). Table 4 shows the lineal regression analysis, where the myth questionnaire score was associated with maternal age and years of schooling, and the knowledge questionnaire score was associated with age and number of pregnancies.

### 3.1. Implementing Myths or not Acting on Knowledge

Most participants, although they knew that caffeine could be harmful, consumed it. Table 5 shows how, in general, participants with and without correct answers had similar practices in some items. These results are an example that having correct or adequate knowledge is no guarantee of putting this knowledge into practice.

### 3.2. Probability of Acting on Myths and not Practicing Knowledge

The general linear model found nonsignificant interactions between factors. For the myths questionnaire, age group and occupation F = 3271, *p* = 0.07, age group and marital status F = 1393, *p* = 0.238, and occupation and marital status F = 0.103, *p* = 0.74. For the knowledge questionnaire, age group and occupation F = 0.409, *p* = 0.07, age group and marital status F = 0.260, *p* = 0.60, occupation and marital status F = 0.696, *p* = 0.40, and age group, occupation, and marital status F = 0.038, *p* = 0.84. Some sociodemographic variables were associated with the probability of practicing, or not, any myth or knowledge, as can see in Table 6. Being an adolescent was the most common among the sociodemographic variables, followed by a low level of education.

Logistic regression models included education, marital status, and place of work, age group, and educational lag. Only variables with a *p*-value < 0.05 are shown. 95% CI, 95% confidence interval. Note the items ‘The mother requires an adequate increased energy intake during pregnancy and nursing” and “During the first six months of life, breast milk is the only food a baby requires” are not shown because no variable was statistically associated.

## 4. Discussion

### 4.1. Myths about Nutrition and Diet during Pregnancy

The present study is one of the first to provide information on the myths and knowledge of pregnant Mexicans on nutrition and diet during pregnancy and breastfeeding. The myths studied here reflect some of the scientifically unfounded beliefs of the socio-cultural context of women in low to lower-middle socioeconomic statuses in Mexico City and the surrounding metropolitan area. In our sample of pregnant Mexicans, 91% of the participants had at least one belief; this figure is close to the 90% reported for Italian adult women [14].

Certain variables were more closely related to myths and knowledge, reflecting previous reports in the literature. For example, in our study, being married was a risk factor for believing that “you have to eat for two” during pregnancy. A previous publication showed that women’s partners encouraged them to eat more during pregnancy because they believed it would be easy for the women to lose weight in the postpartum period [30]. In our study, lower educational attainment was associated with holding and acting upon myths, and the belief that “not satisfying cravings leaves a mark on the body of the newborn” was most frequent among our adolescent participants. Another myth associated with a low socioeconomic level was “you cannot exercise during pregnancy,” as reported previously by Weir Z. et al. in a qualitative study of the myths of overweight and obese pregnant women [31]. Especially in our population, a lower education level was associated with being a homemaker. Indeed, close to 80% of our participants were solely dedicated to being a homemaker with no outside economic activity.

### 4.2. Myths about Nutrition and Diet during Breastfeeding

A large proportion of our study population believed that the consumption of *atole* improves the production of breast milk, reflecting a generalized Mexican belief [32]. However, high *atole* intake also implies excessive sugar intake as well, which would, in turn, increase the risk of gaining more weight or retaining the weight gained during pregnancy. Regarding beer intake, there is evidence that alcohol-free beer during pregnancy increases the antioxidant activity in breast milk due to polyphenols. However, Mexican women drink regular alcoholic beer for enhancing breast milk production—not for the antioxidants [33]. This kind of belief during breastfeeding is not unique to Mexico. For instance, African and Asian immigrants in Canada believe that mothers should eat sweets to increase breast milk production [34].

In our data, 32% of our sample believed the myth that beer consumption increases milk production. Nevertheless, we would like to highlight that 36% of the participants would act upon this myth. This could clarify the relevance of believing and practicing certain myths to achieve well-being, even though without scientific support. Similar findings are in line with those of Mennella and Beauchamp [21] and Koletzko and Lehner [35]. Researches who refer to traditional wisdom not only in Bavaria but also in many other areas of the world, such as Mexico, claim that moderate beer consumption can be beneficial for the onset of breastfeeding and for increasing the success of breastfeeding [33].

In general, we could observe that the age and education years explained the myth scores, especially for certain myths. For example, younger participants in our study, women with the lowest levels of education and lower socioeconomic status, and fewer previous pregnancies were most likely to believe that “a frightened or angry mother should not nurse a baby” and “drinking *atole* or beer enhances maternal milk production”, as showed by Swigart et al., in low-income communities in Mexico [36]. On the other hand, it has been reported that older mothers held fewer myths; the reason may be due to that they have more experience [10]. Besides, the stages of pregnancy and breastfeeding could encourage mothers to have better nutrition and diet.

### 4.3. Knowledge

Two aspects of incongruence between knowledge and actions have been previously described: folic acid supplementation and caffeine. In our case, almost 80% of our sample recognized that “folic acid intake should begin before—not only during—pregnancy.” However, almost one third did not take a folic acid supplement; similar findings were described in women from the Middle East [37]. Other studies reported that most women know that they should take folic acid but do not know exactly how folic acid could benefit them and their babies (e.g., prevention of neural tube defects) [38,39]. In our study, being an adolescent and not having any higher education were both associated with consuming coffee despite reporting knowledge that caffeine can cause premature labor and delivery. A study in Canada showed that pregnant adolescents, usually in high school, knew about the harmful effects of caffeine, but not the quantity that could be consumed without producing these effects [40].

One aspect of knowledge that was not able to be measured as a behavior was the connection between gestational diabetes and type 2 diabetes. We did notice, and would like to highlight, that close 20% of all our participants did not know about this increased risk. Other research found that this lack of knowledge may prevent women from seeking professional advice for improving their diet and lifestyle on time to delay or prevent the onset of type 2 diabetes mellitus [41].

### 4.4. Incongruence between Beliefs, Knowledge, and Practice

Whereas 97% of our participants referred the need for a healthy diet and lifestyle during their pregnancy to avoid future diseases for themselves and their children, only one-third had implemented this knowledge through associated behaviors. Even though a significant (16%) percentage of mothers did not hold the belief that pregnant women “have to eat for two” or satisfy cravings, they actually ate for two and satisfied all their cravings. A previous study reported that overeating during pregnancy is associated with a lack of exercise, meaning that some women that eat for two could be at risk of gaining excessive gestational weight [42].

Another common belief held by our participants was that it is impossible to recover their pregestational weight after childbirth. This myth was prevalent in one-fifth of all participants, with fewer education years being associated with a higher probability of holding this belief. Our finding was similar to that published by Ledoux T et al. [43], who reported that women who understood exactly how much weight they should gain during pregnancy followed guidelines more closely and were more likely to achieve adequate gestational weight.

### 4.5. Myths and Newborn Weight

We observed that the weight of the newborn was associated with beliefs in certain myths. However, the average weight of our newborns was within the normal parameters according to national and international standards, except for those reported in a rural region of Mexico [24]. This fact could be, probably, because Mexico currently faces a severe problem of overweight and obesity, and perhaps the effect of malnutrition may be so changing the myths about the weight of the newborn [22,25].

### 4.6. Implications

Generally, pregnant and breastfeeding women tend to modify their diet according to the myths transmitted from generation to generation in an attempt to improve their health and that of their children. However, they often implement these modifications without the guidance of a nutrition or health specialist, as reported by Pinheiro [44]. This situation represents a window of opportunity for perinatal health-care professionals, who, through adequate programs of intervention, can improve the nutrition and gestational weight gain of their patients, especially adolescents. The attention to the nutrition and diet of pregnant and breastfeeding women is a weak point of health-care systems in Latin American [45].

It is of utmost importance for healthcare professionals (including dieticians) to assume their role in modifying myths surrounding diet and nutrition during pregnancy and breastfeeding. A successful effort requires understanding the myths of women in a particular society and culture. Finally, it is essential to highlight that nutritional guidance for pregnant women will require not only the transmission of knowledge but also the interpretation of information within the context of certain culturally rooted beliefs [46].

On the other hand, the knowledge with the most incorrect answers was the following: “For a successful breastfeeding, a baby must catch the nipple and use adequate suction to feed”; this fact can probably result in mothers not achieving adequate exclusive breastfeeding [47]. Another of the findings observed in our study refers to an adequate knowledge about the amount of liquids that should be consumed during breastfeeding. Therefore, there are two problems in a single population group: inadequate knowledge about the correct breastfeeding technique and the adequate amount of fluids that should be consumed during breastfeeding. These two problems can be associated negatively with successful breastfeeding, which has a negative impact on the mother’s health like that of her child [48]. Our results are in accordance with that reported by Martinez H and Zhou Y et al., [49,50] who have reported that only 14% to 46% of the women studied consumed an adequate amount of water during breastfeeding, and that approximately half of their children were fed milk formula.

### 4.7. Strengths and Limitations

As this is a cross-sectional study, the associations found do not imply causality. Nevertheless, this study offers a broad panorama of the interaction between beliefs and knowledge that condition the ability and willingness of pregnant adolescents and adults to adopt a healthy diet. This new information described is the first attempt at exploring patterns of beliefs, knowledge, and practice to help pregnant women practice an adequately nutritious diet. Therefore, health professionals should fully comprehend the influence that beliefs have on nutrition practice during pregnancy and breastfeeding. A comprehensive approach and efficient communication surrounding this issue could reduce the gap between scientific recommendations and daily eating practices of pregnant and breastfeeding women.

## 5. Conclusions

Among a sample of pregnant Mexicans, almost all held at least one myth about nutrition and diet during pregnancy and breastfeeding. Younger women held myths at a higher frequency than older women, and being adolescent was associated with holding myths. All participants in our study showed inconsistency between not believing in certain myths but acting upon them. Although most of our participants had adequate knowledge of diet and nutrition in pregnancy and breastfeeding, they did not act on this knowledge and practice it.

## Figures and Tables

**Table 1:** Scores of the questionnaires applied to 1017 pregnant women.

Myths			Knowledge		
Score	*n*	%	Score	*n*	%
0	5	0.5	3	1	0.1
1	37	3.6	4	3	0.3
2	71	7.0	5	16	1.6
3	134	13.2	6	60	5.9
4	222	21.8	7	172	16.9
5	241	23.7	8	305	30.0
6	217	21.3	9	296	29.1
7	90	8.8	10	164	16.1
Total	1017	100	Total	1017	100

**Table 2:** General characteristics of the participants, according to median myths and knowledge score.

	Myths			Knowledge		
Characteristics	Below Median * n = 469	Above Median * n = 548	*p*-Value	Below Median * n = 80	Above Median * n = 937	*p*-Value
Mother ^a^ Mean (standard deviation)						
Age (yrs.)	23 (7)	27 (8)	0.001	27 (9)	25 (8)	0.116
Pregestational weight (kg)	57 (12)	61 (13)	0.001	59 (12)	59 (13)	0.979
BMI pregestational	23 (5)	25 (4)	0.001	24 (4)	24 (4)	0.725
GWG (kg)	12 (6)	11 (7)	0.238	11 (7)	11 (6)	0.647
Pregnancies (#)	2 (1.0)	1 (1.3)	0.001	1.7 (1.0)	1.8 (1.2)	0.502
Newborn ^a^ Mean (standard deviation)						
Gestational age (weeks)	38 (2)	38 (2)	0.192	38 (2)	38 (2)	0.161
Length (cm)	48 (3)	48 (3)	0.085	48 (4)	48 (3)	0.097
Weight (g)	2839 (600)	2963 (552)	0.001	2835 (580)	2913 (580)	0.261
Sociodemographic data ^b^ Frequency (%)						
Education (yrs.)	9 (9–12)	12 (9–14)	0.001	12 (9–12)	12 (9–12)	0.594
Socio-economic (level)	2 (1–2)	2 (2–2)	0.001	2 (1–2)	2 (1–2)	0.434
Educational lag						
Yes	345 (74)	336 (61)	0.001	52 (65)	630 (67)	0.384
No	124 (26)	212 (39)		28 (35)	307 (33)	
Place of work Homemaker	388 (83)	422 (77)	0.012	65 (82)	743 (80)	0.333
Outside the home	80 (17)	127 (23)		14 (18)	192 (20)	
Socioeconomic level						
Middle	52 (11)	133 (24)	0.001	14 (17)	171 (18)	0.867
Low	417 (89)	415 (76)		66 (83)	766 (82)	

^a^*p*-value from Student’s t-test, ^b^ Pearson Chi^2^; GWG, Gestational Weight Gain; yrs., years; BMI, body mass index. * Above average or below average according to median score.

**Table 3:** Maternal and neonatal variables associated with belief in dietetic myths during pregnancy and breastfeeding, mean (standard deviation).

		Age (yrs.)	Education (yrs.)	Socio-Economic Level	Number of Pregnancies	Baby Weight (g)	BMI p	GWG (kg)
Pregnancy								
*You must eat for two during pregnancy*	Correct (*n* = 719, 71%)	26.7 (8) ^a^	11.7 (2.6) ^a^	2.1 (0.8) ^a^	1.93 (1.2) ^a^	2937 (587) ^b^	25 (5) ^a^	11.0 (7.1)
	Incorrect (*n* = 298, 29%)	22.8 (8)	9.8 (2.1)	1.7 (0.6)	1.63 (1.1)	2831 (556)	22 (3)	11.6 (7.4)
*Not satisfying cravings leave a mark on the infant’s body*	Correct (*n* = 811, 80%)	23 (7.7) ^a^	11.4 (2.6) ^a^	2.0 (0.8) ^a^	1.9 (1.2) ^b^	2927 (563) ^a^	24 (5) ^b^	11.2 (7.3)
	Incorrect (*n* = 206, 20%)	26.2 (8.2)	10 (2.2)	1.7 (0.72)	1.6 (1.1)	2827 (637)	23 (5)	11.2 (6.7)
*Vomiting cannot be controlled during pregnancy*	Correct (*n* = 399, 39%)	27 (8.1) ^a^	11.6 (2.6) ^a^	1.9 (0.78)	1.93 (1.2) ^b^	2942 (565)	25 (5) ^b^	11.3 (7-6)
	Incorrect (*n* = 618, 61%)	24.6 (8.1)	10.8 (2.5)	2.0 (0.86)	1.78 (1.1)	2883 (589)	24 (5)	11.1 (6.9))
*You cannot exercise during pregnancy*	Correct (*n* = 825, 81%)	25.8 (8.2) ^b^	11.3 (2.6) ^a^	2.0 (0.85) ^a^	1.85 (1.2)	2925 (577) ^a^	24 (5)	11.09 (7.1)
	Incorrect (*n* = 192, 19%)	24.2 (8.0)	10.2 (2.3)	1.72 (0.58)	1.82 (1.1)	2825 (589)	24 (5)	11.8 (7.8)
*It is impossible to recover one’s pre-baby weight after childbirth*	Correct (*n* = 818, 80%)	25.6 (8.2)	11.3 (2.6) ^a^	1.96 (0.48) ^b^	1.8 (1.1)	2926 (574)^b^	24 (5)	10.9 (7.1) ^b^
	Incorrect (*n* = 199, 20%)	25.1 (8.1)	10.5 (2.4)	1.89 (0.69)	1.9 (1.2)	2823 (598)	24 (5)	12.3 (7.4)
Breastfeeding								
*Drinking “atole” or beer enhances breast milk production*	Correct (*n* = 674, 66%)	26.6 (8.3) ^a^	11.4 (2.6) ^a^	2.0 (0.8) ^a^	1.9 (1.2) ^a^	2915 (575)	24 (5) ^b^	10.9 (7.5)
	Incorrect (*n* = 343, 34%)	23.5 (7.4)	10.7 (2.4)	1.8 (0.7)	1.6 (1.0)	2889 (590)	25 (5)	11.8 (6.5)
*A frightened or angry mother should not nurse her baby*	Correct (*n* = 360, 36%)	26.8 (8.1) ^a^	11.7 (2.8) ^a^	2.07 (1.3) ^a^	2.03 (1.3) ^a^	2917 (584)	24 (5)	11.5 (7.7)
	Incorrect (*n* = 657, 64%)	24.9 (8.1)	10.8 (2.7)	1.88 (0.7)	1.74 (1.1)	2901 (578)	24 (5)	11.0 (6.9)

Mean (standard deviation). ^a^ *p* ≤ 0.001 Student’s t-test, ^b^ *p* < 0.050 Student’s t-test, BMIp = pregestational body mass index, GWG = Gestational Weight Gain. This table shows only the variables that showed a *p*-value < 0.05; the missing variables are not shown because statistical significance was not found.

**Table 4:** Myths and knowledge scores and their covariables.

	Mean	Β	Standard Error	95% CI	*p*-Value
Myths *					
Score	4				
Age		0.025	0.007	0.011, 0.040	<0.001
Number of pregnancies		0.069	0.043	−0.015,0.153	0.117
Socioeconomic level		0.060	0.066	−0.070, 0.189	0.365
Years of schooling		0.174	0.020	0.136, 0.213	<0.001
Knowledge ^§^					
Score	8				
Age		−0.017	0.006	−0.028, −0.005	0.005
Number of pregnancies		0.077	0.036	0.002, 0.148	0.033
Socioeconomic level		0.056	0.056	−0.052, 0.164	0.310
Years of schooling		−0.002	0.017	−0.029, 0.031	0.927

Linear regression. 95% CI, confidence interval. * R^2^ 0.150, Adjusted R^2^ 0.147. **^§^** R^2^ 0.009, Adjusted R^2^ = 0.005. Note: all variables included in the regression models are presented.

**Table 5:** Distribution of questionnaire responses by women who practice myths and do not act on scientific knowledge, frequency (%).

Answer	Correct	Incorrect	*p*-Value
Practice a Myth			
• Pregnancy			
You must eat for two during pregnancy (*n* = 263)	113 (43)	150 (57)	0.001
Not satisfying cravings leave a mark on the infant’s body (*n* = 541)	429 (79)	112 (21)	0.395
Vomiting cannot be controlled during pregnancy (*n* = 30)	11 (37)	19 (63)	0.973
You cannot exercise during pregnancy (*n* = 635)	494 (78)	141 (22)	0.001
• Breastfeeding			
Drinking *atole* or beer enhances breast milk production (*n* = 144)	70 (49)	74 (51)	0.001
A frightened or angry mother should not nurse her baby(*n* = 107)	34 (32)	73 (68)	0.407
Not practice knowledge			
• Pregnancy			
Caffeine consumption provokes premature birth (*n* = 353)	302 (86)	51 (14)	0.002
Folic acid intake should begin before—not only during—pregnancy (*n* = 767)	469 (61)	298 (39)	0.005
A healthy diet and lifestyle during pregnancy prevents future diseases in a child (*n* = 636)	598 (94))	38 (6)	0.232
• Breastfeeding			
A mother should consume about 3 liters of water per day while nursing (*n* = 688)	432 (63)	256 (37)	0.734
Mothers require adequate increased energy intake during pregnancy and nursing(*n* = 1003)	640 (64)	363 (36)	0.286
During the first six months of life, breast milk is the only food a baby requires (*n* = 930)	868 (93)	62 (7)	0.373

Comparison of frequencies by Pearson Chi^2^. Note: some questions were excluded from analysis because they were not directly actionable. For example, there is no practical way to act out how gestational diabetes evolves into type 2 diabetes.

**Table 6:** Variables associated with acting on myths and not practicing knowledge.

	OR	95% CI	*p*-Value
Myths			
You must eat for two during pregnancy			
*• Being an adolescent*	1.76	1.26–2.44	0.001
*• Being married*	1.47	1.11–1.95	0.007
Not satisfying cravings leave a mark on the infant’s body			
*• Being an adolescent*	2.41	1.78–3.26	0.001
You cannot exercise during pregnancy			
*• Being an adolescent*	1.59	1.16–2.18	0.003
*• Low socioeconomic level*	1.41	1.01–1.95	0.038
Drinking *atole* or beer enhances breast milk production			
*• Being single*	2.07	1.38–3.09	0.001
A frightened or angry mother should not nurse her baby			
*• Being an adult*	2.61	1.35–5.04	0.004
Knowledge			
Caffeine consumption provokes premature birth			
*• Being an adolescent*	1.42	1.05–1.91	0.022
*• High school or below*	1.38	1.04–1.84	0.025
Folic acid intake should begin before—not only during—pregnancy			
*• High school or below*	1.53	1.14–2.05	0.004
A healthy diet and lifestyle during pregnancy prevents future diseases in a child			
*• Being a homemaker*	1.26	0.92–1.73	0.138
A mother should consume about 3 liters of water per day while nursing			
*• Being an adolescent*	1.86	1.22–2.82	0.003
*• High school or below*	1.38	1.00–1.91	0.046

## Abbreviations

CI	Confidence Interval
BMI	Body Mass Index
OR	Odds Ratio
